# Tower of Babel or Lighthouse? The State of Research on Neuroelectric Correlates of Human Sexuality: A Response to the Commentaries

**DOI:** 10.1007/s10508-022-02496-0

**Published:** 2022-12-08

**Authors:** Andreas Mokros, Elmar Habermeyer, Timm B. Poeppl, Pekka Santtila, Anastasios Ziogas

**Affiliations:** 1 Faculty of Psychology, FernUniversität in Hagen, 58084 Hagen, Germany; 2 Department of Forensic Psychiatry, University Hospital of Psychiatry Zurich, Zurich, Switzerland; 3 Department of Psychiatry and Psychotherapy, University of Regensburg, Regensburg, Germany; 4 Institute for Social Development, New York University Shanghai, Shanghai, China; 5 Faculty of Psychology, UniDistance Suisse, Brig, Switzerland

As authors of the Target Article (Ziogas et al., 2020), we would like to thank all five colleagues who were so kind as to comment on our article. We are feeling flattered by their kindness, impressed by their thoroughness, and inspired by their invaluable suggestions on how to move on from here.

First, we would like to address each Commentary in turn, in alphabetical order, trying to summarize its main gist, and reflect upon our review accordingly. Second, in doing so, we want to add a few thoughts on the backdrop against which the “Tower of Babel” was erected, as Pfaus (2021) put it. Third, we would like to emphasize some particular recommendations on behalf of the commentators on how our field might move forward productively.

Huberman (2021) focused on the heterogeneity of stimulus material as indicated by our review. As a working definition, Huberman considered as sexual cues those kinds of stimuli “that have the capacity to evoke sexual arousal or interest.” This broad take summarizes the dilemma well as nearly all stimuli can obtain a sexual quality through coupling with unconditional reactions, excitation transfer, imagination, and several other processes. The almost endless list of paraphilias (and associated stimuli) described by Money (1986) creates the impression that being sexual is not some innate quality of a cue, but a latent meaning ascribed to it by different individuals. Far from being unequivocal, a pair of high heels may be a mundane piece of clothing to one person and an exciting ticket to bliss for another. This stance is somewhat captured by the words of Schopenhauer (1851/1892): “The same external events or circumstances affect no two people alike; even with perfectly similar surroundings every one lives in a world of his own” (p. 4).

Apart from the almost intangible (or at least highly idiosyncratic) nature of a stimulus as sexual, Huberman (2021) pointed at the confounding quality of different stimulus properties: How should we present sexual stimuli depicting human beings without showing faces, bodies, postures, expressions, clothing (or the lack thereof) at the same time? We concur with Huberman that our review possibly identified the heterogeneity but did not provide much in terms of a direction out of this jungle of stimulus options. Clearly, the evaluation of the stimuli by the participants themselves (in addition to recording their physiological responses through, say, event-related potentials [ERPs]) seems to be the most viable option and was espoused by one other Commentary as well (Ristow & Kärgel, 2021).

With respect to one question that Huberman (2021) distilled from our review—“[Can] we conclude that neural responses differentiating sexual and nonsexual stimuli are specific to processing sexual stimuli?”—some of us (AZ, EH, and AM) attempted to provide an answer with a study using ERPs (Ziogas et al., 2022). Comparing 40 heterosexual and 40 gay men, half of whom were instructed to mimic the responses of the other sexual orientation, we noted a difference in the positive slow wave (PSW) depending on match between actual sexual orientation and stimulus content. Contrary to expectation, however, the PSW was attenuated (not accentuated) in trials containing images matching the individuals’ sexual orientation. According to expectation, however, more explicit images (i.e., pictures of nude individuals) elicited more pronounced early posterior negativity (EPN). Incidentally, the ERP study by Ziogas et al. (2022) included a rating of the stimuli in terms of valence, arousal, and sexual attractiveness by the participants themselves, thus incorporating a recommendation made in one Commentary (Ristow & Kärgel, 2021).

Huberman (2021) also mentioned the dynamic nature of the sexual arousal response that entails attentional and motivational processes, among others. Given different stages of sexual arousal (Singer, 1984) or stimulus appraisal (Janssen et al., 2000), “the novelty [or] the taboo content” (Huberman, 2021) may be crucial for any electrophysiological signals detected. After all, longer response latencies in response to sexual stimuli have been explained as an effect of hesitation on behalf of the participant (i.e., sexual content induced delay; Geer & Bellard, 1996; Geer & Melton, 1997). Alternatively, in viewing time tasks, longer response times could be explained with cognitive rather than affective processes (i.e., judging the stimuli from the perspective of a person with this or that sexual orientation; see, e.g., Imhoff et al., 2012). We might add that the stage of the sexual arousal response as well as emotional states (such as disgust; see, e.g., Hinzmann et al., 2020) likely interact with the appraisal of stimuli and, potentially, also shape the electrophysiological signals emitted.

Finally, Huberman (2021) addressed those parts of our review that dealt with between-subjects designs in which individuals with some sort of paraphilia were compared with other persons who do not share that particular paraphilia. In this regard we understand Huberman’s position as recommending focusing on within-subject processes rather than on between-group differences. It seems plausible that Huberman is right in assuming that the former (i.e., studying general processes at the individual level) will prove more fruitful. We might add that with the so-called reliability paradox in mind, between-group differences may be too difficult to detect in this area. According to Hedge et al. (2018), the reliability paradox indicates that well-established tasks from cognitive psychology are not appropriate for individual differences research because their generic effect is so large and common as to absorb any potential associations with individual differences.

In his Commentary, Komisaruk (2020) highlighted both a particular limitation and a potential threat. In terms of a limitation, Komisaruk pointed out that, despite incorporating 255 empirical studies, our review was narrow in the sense of not straying away from the field of neuroelectric studies. Komisaruk indicated that we “ignore[d] functional magnetic resonance imaging (fMRI) and positron emission tomography (PET) studies of sexual arousal and orgasm” which would have afforded the advantage of much more spatial detail and more accurate topical localization. Clearly, it was not our conviction that neuroimaging research is irrelevant. This is evidenced, for instance, by systematic reviews on neuroimaging findings regarding sexual arousal by members of our group (Poeppl et al., 2014, 2020). Several reviews on fMRI and human sexuality have already been published with different thematic foci (e.g., Joyal et al., 2007; Stoléru et al., 2012), whereas neuroelectric studies had not been summarized in the literature previously. More to the point, we consider electrophysiology and brain imaging as complementary techniques. Ideally, the two methods should be combined in a suite of experiments on the same participants using the same set of stimuli. Reviewing the extant literature from both fields simultaneously would possibly have been beyond the publishing limits of the *Archives of Sexual Behavior*. It surely would have been beyond our capacity. Moreover, we intended to highlight the potential of electrophysiological methods in the domain of research on human sexuality. In this sense, we would like to cite the opinion of the third commentator, Pfaus (2021), that the different strengths of both types of techniques (i.e., temporal v. spatial resolution) “make it extremely difficult to relate findings even from the same brain regions.”

The second concern raised by Komisaruk (2020) refers to an ethical issue, not a methodological one: The neutral stance we took toward the potential legal ramifications of electrophysiological research on sexual arousal in general and on paraphilias in particular. While it is our conviction that empirical findings should strictly be separated from personal opinion, we may now use the opportunity of voicing our opinion here, within the somewhat different genre of debate and commentary: Although the techniques reviewed have, in part, an astounding level of sophistication, this does not mean that their outcome is necessarily true or even more useful to medical or even legal questions than integrative expert evidence.

In a similar regard, Morse (as cited in Buchen, 2012, p. 306) coined the term of “brain overclaim syndrome” in order to illustrate the problem that some neuroscientists attach too much weight to machines recording brain activity and structure when applied to legal matters because neuroscience data may appear to be harder science than psychological or psychiatric assessment. More specifically, Morse referred to the fallacy of assuming causation when the neuroscience data merely demonstrate correlation. The case of schizophrenia may serve as a sobering guiding light in this context: Although schizophrenia accounts for the majority of cases considered not guilty for reason of insanity, and even though schizophrenia has been subject to most intense scrutiny and biomedical research during the last century, there still is neither a reliable biomarker available nor would the results from, say, fMRI replace the comprehensive assessment of expert witnesses in terms of legal culpability. The plasticity of the brain, interindividual differences in anatomy and structure, as well as a lack of findings on temporal, cross-situational robustness (i.e., reliability) preclude the sole use of electrophysiological or brain imaging data. Nevertheless, they may serve as additional pieces of information within a comprehensive assessment. Their admissibility in court differs with legal system (e.g., adversarial vs. non-adversarial) and across countries (see, for instance, Guillen-Gonzalez et al., 2019).

In his Commentary, Pfaus (2021) not only found the most friendly words to describe our work but identified a reassuring core finding: According to him, it was “remarkable […] that there are *any* commonalities in the findings” (italics in original) given the plethora of methods, stimuli, samples, designs, measurements, and analyses used within the domain of neuroelectric activity pertaining to sexuality. Instead of complaining about that we still know so little, Pfaus maintains that we do know something despite all that variability. Picking up his line of argument—and his emphasis on the lack of any “agreed-upon basic methodolog[y] in […] brain activation or stimulation studies”—we would like to connect the findings with another unsavory but healthy debate: The ramifications of the so-called replication crisis in both medicine (Ioannidis, 2005) and psychology (Open Science Collaboration, 2015) as well as the movement toward open and transparent research practice as a promising remedy. Borrowing findings from a related field, namely brain imaging research, highlights this phenomenon: Poldrack et al. (2017) identified about 70,000 different possible workflows for pre-processing and analyzing corresponding data. Similarly, Botvinik-Nezer et al. (2020) found that there was not a single best practice but high variability between 70 workgroups when analyzing the same set of brain imaging data. Recently, this phenomenon that is oftentimes referred to as akin to a garden of forking paths has also been demonstrated for ERP research (Šoškić et al., 2022). (Originally, the notion of the “garden of forking paths” [Gelman & Loken, 2013, p. 1; 2014] described the increase in the risk of committing a type I error if data-analytic decisions are made in view of the data at hand). In that sense, a move toward pre-registration of hypotheses, the availability of datasets for re-analysis, and transparency in terms of materials used and methods applied may not only ameliorate the problems identified in the wake of the so-called replication crisis but lead to more productive outcomes through standardizing procedures and methods, making findings more easily compatible and comparable. Therefore, we would encourage Pfaus’s idea of a symposium at the International Society of Sex Research devoted to finding consensus and agreeing on standard procedures.

Among other important issues and apart from addressing methodological heterogeneity, Pfaus (2021) also stressed the importance of sociocultural conditions (and the change thereof) with respect to sex research in general and the neuroelectric domain in particular. We fully agree that what is regarded as attractive and sexually arousing is not independent of developments at the level of society at large. The availability of sexually arousing images as well as their type has greatly changed since the mid-twentieth century. We completely agree that the prospect of further change in taste and preference endangers the long-term suitability of standardized stimuli. Again, the notion of stimulus ratings on behalf of the participants themselves provides a potential remedy for this drawback.

Ultimately, Pfaus (2021) as well as the final comment by Ristow and Kärgel (2021) pointed at the potential of studies including transcranial direct current stimulation (tDCS), the latter explicitly mentioning a study (Pezzoli et al., 2021) that three of us (AZ, AM, and PS) were involved with. The appealing promise of tDCS is that brain activation can be modulated in a reversible and non-invasive manner, thus opening up the possibility of experimental interventions that target particular areas specifically. With regard to the study by Pezzoli et al., however, we have to concede that the expected tDCS effect on attentional bias for child stimuli in pedophilic patients could not be ascertained. These results, however, may be negative only due to the specific brain stimulation technique employed rather than a general ineffectiveness of non-invasive brain stimulation in modulating sexual behavior. This notion would be corroborated by our own observation that repetitive transcranial magnetic stimulation (rTMS) is a potential tool to reduce sexual arousal (Schecklmann et al., 2020), while tDCS does not exert a significant effect in an otherwise identical experimental setting (Sakreida et al., 2022). Moreover, in our study with pedophilic patients, we might simply have missed the “hotspot” by targeting the left dorsolateral prefrontal cortex (DLPFC), given evidence that high-frequency rTMS of the right but not left DLPFC could significantly reduce subjective sexual arousal (Schecklmann et al., 2020). Although these preliminary studies leave many questions open for debate and even raise further questions, we agree with Ristow and Kärgel (2021) that non-invasive brain stimulation holds the potential to “open up a potential for the development of therapeutic intervention” also in sexual disorders.

Turning to the fourth and last Commentary by Ristow and Kärgel (2021) more thoroughly, the term biomarker is at the center of attention. As Ristow and Kärgel convincingly argued, the identification of a single indicator seems highly unlikely. Rather, they suggested that neuroelectric methods may complement other (e.g., neuropsychological or brain imaging) data in this regard. At the same time, Ristow and Kärgel took up the concern voiced by Komisaruk (2020) on the potential misuse of apparently objective physiological indicators when applied in the legal domain. In this respect, Ristow and Kärgel mentioned the risk of false-positive decisions—a danger that we would like to emphasize. In a transcranial magnetic stimulation study, we could demonstrate that sexual motivation was reflected by stimulus-dependent motor cortex excitability (Schecklmann et al., 2015). More specifically, our data suggested that amplitudes of motor-evoked potentials (MEPs) in response to visual sexual stimuli significantly depended on (the match between stimulus type and) sexual orientation. Although motor cortex excitability might thus be considered a “biomarker” of sexual orientation and perhaps also be good as a biological indicator of atypical sexuality, this measure does not qualify as a basis for important medico-legal decisions due to its high intra- and interindividual variability.

Moreover, Ristow and Kärgel (2021) highlighted the heterogeneity of samples comprising individuals with paraphilias or with a history of corresponding offenses. Such samples likely include different subtypes or variants of different etiology. We agree, as Ristow and Kärgel pointed out, that aspects like impulsivity or inhibitory control may be more relevant for one subtype than for another—a differentiation that likely influences treatment efficacy as well.

In sum, as authors of the Target Article, we would like to emphasize our gratefulness to the five commentators. We hope that the systematic review of the extant literature as laid down in the Target Article as well as the productive debate through open peer commentary shows one thing: That decades of research on neuroelectric correlates of human sexuality were not a forlorn enterprise but a fruitful one. The path taken may not have been a straightforward one but a meandering one. Nevertheless, we hope that the lessons learned from the previous findings turn the body of knowledge into a lighthouse, not into a Tower of Babel. Put succinctly, standardization (of stimuli and procedures), stronger theoretical and methodological rigor (in applying the principles of open science), as well as comprehensiveness (in combining different approaches like ERPs and brain imaging techniques) are viable options to improve the current state of research.
